# Comparison of Bleeding Pattern and Quality of Life Before and After the Insertion of a Levonorgestrel Intrauterine System for Heavy Menstrual Bleeding: A Seven-Year Review

**DOI:** 10.7759/cureus.36142

**Published:** 2023-03-14

**Authors:** Mukta Agarwal, Smita Singh, Shivangni Sinha, Hemali H Sinha

**Affiliations:** 1 Obstetrics and Gynecology, All India Institute of Medical Sciences, Patna, Patna, IND

**Keywords:** menorrhagia, lng-ius, sf-36, pbas, mmas, levonorgestrel, quality of life, heavy menstrual bleeding

## Abstract

Background

This study aimed to examine the role of a levonorgestrel intrauterine system (LNG-IUS) in the treatment of heavy menstrual bleeding (HMB) regarding improvements in bleeding patterns and quality of life (QOL) and determine the reason for its failure or withdrawal from treatment in some patients.

Methodology

This retrospective study was conducted in a tertiary care center in eastern India. A seven-year assessment, with both qualitative and quantitative assessments, of the effect of LNG-IUS in women with HMB was performed using the Menorrhagia Multiattribute Scale (MMAS) and Medical Outcomes Study 36-Item Short-Form Health Survey (MOS SF-36) score as a tool to assess the QOL, and the pictorial bleeding assessment chart (PBAC) to assess bleeding patterns. The study population was divided into the following four groups based on duration: three months to one year, one to two years, two to three years, and more than years. The continuation, expulsion, and hysterectomy rates were evaluated.

Results

The mean MMAS and MOS SF-36 scores increased significantly (p < 0.05) from 36.73 ± 20.40 to 93.72 ± 14.62 and 35.33 ± 6.73 to 90.54 ± 15.89, respectively. The mean PBAC score decreased from 176.36 ± 79.85 to 32.19 ± 63.87. In total, 348 women (94.25%) continued the LNG-IUS, and 3.44% had uncontrolled menorrhagia. Furthermore, at the end of seven years, the expulsion rate was 2.28% due to adenomyosis and pelvic inflammatory disease, and the hysterectomy rate was 5.75%. In addition, 45.97% and 48.27% of the participants had amenorrhea and hypomenorrhea, respectively.

Conclusions

LNG-IUS improves bleeding and QOL in women with HMB. In addition, it requires less skill and is a non-invasive and nonsurgical option, which should be considered first.

## Introduction

Heavy menstrual bleeding (HMB), a common gynecological complaint among women in their reproductive ages, comprises 12% of all gynecological visits [1]. Globally, evidence suggests that 4-51.6% of women experience HMB [2]. Furthermore, in India, its prevalence is 17.9% [3].

Traditionally, HMB, previously known as menorrhagia, was measured quantitatively using the number of pads, duration of flow, the passage of clots, or fall in hemoglobin level. However, quantitative measurement of HMB was not feasible. Hence, in 2018, the National Institute for Health and Care Excellence (NICE) guidelines stated that “HMB is a very subjective and personal assessment, affecting adversely one’s quality of life (QOL) and should not be adhered to objective measurement only. It should be better considered as excessive menstrual blood loss (MBL) interfering with women’s physical, emotional, social, and material QOL, either alone, or associated with other symptoms.”

Menorrhagia can be treated either medically or surgically. The different medical options include antifibrinolytics, oral contraceptive pills, non-steroidal anti-inflammatory drugs (NSAIDs), systemic progesterone, or gonadotropin-releasing hormone agonists [4]. In contrast, surgical methods include an endometrial ablation, a hysterectomy, or a myomectomy. Although a hysterectomy is definitive, it should be the last treatment option. Among females in the 40-49-year age group, its prevalence was 9.2% based on the national and state-level analysis of the fourth National Family Health Survey (2015-2016) in India. In addition, 55.99% of females underwent a hysterectomy for HMB. Bihar, a state in India, was among the four states with the highest prevalence rates (14.5%) [2].

The levonorgestrel intrauterine system (LNG-IUS), a unique topical treatment for HMB, is recommended as a first-line treatment in women with menorrhagia with no identified pathology, fibroids measuring <3 cm, or suspected or diagnosed adenomyosis. Furthermore, various studies have shown that it has proven and comparative outcomes to other systemic medications and surgery, as well as better QOL [5-8]. However, its popularity is restricted, especially in Asian countries, including India.

Historically, intrauterine devices were developed for contraceptive purposes. However, bleeding problems have evolved as a major reason for the non-acceptance and removal of copper-T. This led to the emergence of LNG-IUS in 1970, first approved in Finland in 1990, and, subsequently, by the US Federal Drug Administration (FDA) for use against HMB in 2015 [9]. It is a T-shaped device that measures 32 mm in both the horizontal arm and vertical body, with a silicon reservoir in its body that contains 52 mg of levonorgestrel and releases 20 µg/day directly into the uterine cavity. Furthermore, it lasts for at least five years. Unlike oral progesterone, there is no peak or trough in the serum LNG level and the overall serum concentration is low. A high level of local progesterone causes endometrial thinning, non-proliferation, and stromal decidualization, known as medical ablation, leading to a decrease in MBL and pain [10].

Menorrhagia has a serious impact on overall health and simultaneously poses an additional economic burden on the family, for which LNG-IUS is a low-cost, non-surgical treatment option. This study aimed to examine its role in the improvement of bleeding patterns and QOL scores before and after its use and the reason for its failure and withdrawal. It is a novel technique to quantify women’s perception of improvement along with the duration of use.

## Materials and methods

This retrospective observational study was conducted in a tertiary care center in Bihar, the third-largest populous state located in eastern India, with a population of 104.2 million. This study was approved by the Institutional Ethical Committee (approval number: AIIMS/Pat/IEC/2021/760). Patients who had undergone an LNG-IUS insertion for at least three cycles for HMB between June 2015 and May 2022 were traced retrospectively from hospital records. They were contacted telephonically and interviewed in person after written informed consent was obtained. Patients who could not be traced or refused to participate were excluded.

This study used three study tools, namely, the Menorrhagia Multiattribute Scale (MMAS) questionnaire, Medical Outcomes Study 36-Item Short-Form Health Survey (MOS SF-36), and Pictorial Bleeding Assessment Chart (PBAC). The MMAS and MOS SF-36 scores were subjective, while the PBAC score was the mean of the objective measurement of HMB.

In-person interviews were conducted with 348 women who underwent LNG-IUS insertion during the study period. They were questioned regarding their bleeding pattern using the PBAC and their QOL using the MMAS and MOS SF-36 before LNG-IUS insertion. Subsequently, all patients were questioned again using the same questionnaires when using the LNG-IUS. Based on the duration of use, they were categorized into the following four groups: three months to one year, one to two years, two to three years, and more than three years. Changes in each score before and after insertion were compared.

The MMAS, developed by Shaw et al. (1998), is a well-accepted menorrhagia-specific questionnaire for subjective assessment of an individual’s QOL. Furthermore, it also measures the outcome of the treatment. It has been proven to have high face validity, a high degree of construct validity, and test-retest reliability with good content and high internal consistency [11,12]. The MMAS scores range from 0 (severely affected) to 100 (unaffected). The effect of HMB on six different domains of life, including practical difficulties, social life, psychological health, physical health and well-being, daily routine, and family relationships, were assessed. Each domain comprised four questions, and each question was scored based on how severally it affected the individual’s QOL. The total score was calculated by adding the scores of each domain.

The SF-36 is a reliable and well-validated tool to assess general well-being over the previous four weeks, as proven by several studies [9,13]. It has eight scales comprising 36 items related to vitality, physical functioning, body pain, general health perception, physical role functioning, emotional role functioning, social role functioning, and mental health. The SF-36 scores range from 0 to 100. The total SF-36 scores were the weighted sum of scores in each section. The higher the score, the greater the QOL.

MBL was scored using the PABC, with scores of 1, 5, and 20 points for lightly, moderately, and completely soaked pads, respectively. Furthermore, 1 point for a small 2 cm diameter clot and 5 points for a large 3 cm clot were added to the total score. A score greater than 100 per menstrual cycle correlated with >80 mL of blood loss, with a sensitivity of 97% in the heavy bleeding population [14]. The objective assessment of blood loss was based solely on the patient’s description of the number of pads used per day (lightly, moderately, or completely soaked), the passage of clots or flooding, or menstrual diary records.

Patients and/or the public were not involved in the design, conduct, reporting, or dissemination of this research.

Statistical analysis

Statistical analyses were performed using SPSS for Windows, Version 25.0 (IBM Corp., Armonk, NY, USA). The results of continuous measurement were presented as mean ± SD. Median (IQR) and categorical values were presented as frequency and percentage. Inferential statistics, such as the Wilcoxon signed-rank test, Kruskal-Wallis test, and Mann-Whitney U test, were used to compare the groups. Statistical significance was set at p-values <0.05.

## Results

Among the 400 LNG-IUS insertions between June 2015 and May 2022, we contacted and interviewed 348 patients. The remaining 52 were either not contactable or refused to participate.

Participants’ ages ranged from 28 to 58 years, with a mean age of 39.74 ± 5.63 years. The majority were in the 31-40-year age group. Based on educational status, 136 (39.1%) were illiterate, 96 (27.6%) had a primary education, and 116 (33.3%) were graduates. Overall, 212 (60.9%), which was a majority of the participants, were homemakers (Table 1).

**Table 1:** Demographic data of the participants.

	Number of cases	Percentage
Age (years)		
21–30	4	1.15
31–40	220	63.22
41–50	108	31.03
51–60	16	4.60
Total	348	100.0
Mean ± SD	39.74 ± 5.63	
Range	28–58	
Education		
Illiterate	136	39.1
Primary	96	27.6
Graduate	116	33.3
Total	348	100.0
Working		
No	212	60.9
Yes	136	39.1
Total	348	100.0

**Figure 1:**
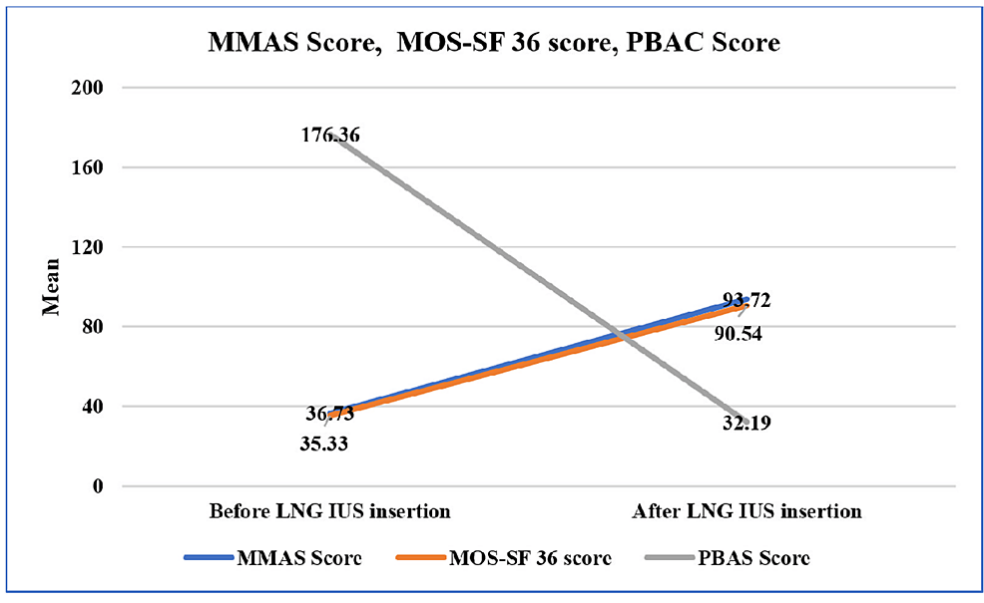
Line diagram depicting the changes in different score means before and after LNG-IUS insertion. MMAS = Menorrhagia Multiattribute Scale; MOS SF-36 = Medical Outcomes Study 36-Item Short-Form Health Survey; PBAS = Pictorial Bleeding Assessment Score; LNG-IUS = levonorgestrel intrauterine system; PBAC = Pictorial Bleeding Assessment Chart

Significant changes in the MMAS, MOS SF-36, and PBAC scores were observed. The MMAS and MOS SF-36 scores increased from baseline, whereas the PBAC scores decreased. At follow-up, the mean MMAS and MOS SF-36 scores increased from 36.73 ± 20.40 to 93.72 ± 14.62 and 35.33 ± 6.73 to 90.54 ± 15.89, respectively. The mean PBAC score decreased from 176.36 ± 79.85 to 32.19 ± 63.87 (Figure 1).

The mean differences were 56.99, 55.21, and 144.16 in the MMAS, MOS SF-36, and PBAC scores, respectively (Table 2).

**Table 2:** The MMAS, MOS SF-36, and PBAC scores. LNG-IUS = levonorgestrel intrauterine system; MMAS = Menorrhagia Multiattribute Scale; MOS SF-36 = Medical Outcomes Study 36-Item Short-Form Health; PBAC = Pictorial Bleeding Assessment Chart

		Range	Mean ± SD	Median (IQR)	P-value
MMAS score	Before LNG-IUS insertion	15.2–64.8	36.73 ± 20.40	26.30 (19.40-59.30)	0.001*
	After LNG-IUS insertion	34.7–100	93.72 ± 14.62	97.90 (93.90-100.0)	
	Difference	12.40–84.80	56.99 ± 20.73	65.30 (35.20-77.70)	
MOS SF-36 score	Before LNG-IUS insertion	20–50	35.33 ± 6.73	35 (30-40)	0.001*
	After LNG-IUS insertion	25–100	90.54 ± 15.89	95 (90-100)	
	Difference	0–75	55.21 ± 13.88	60 (52-65)	
PBAC score	Before LNG-IUS insertion	60–420	176.36 ± 79.85	160 (115-225)	0.001*
	After LNG-IUS insertion	2–300	32.19 ± 63.87	10 (5-30)	
	Difference	20–385	144.16 ± 71.10	136 (95-198)	

Based on education, a significant difference was observed in the MOS SF-36 and PBAC scores. However, the MMAS scores did not differ significantly. Changes in the MMAS and PBAC scores decreased as educational status increased, while changes in the MOS SF-36 score increased as educational status increased. Based on working status, a significant difference was observed in the MMAS and PBAC scores. However, there was no difference in the MOS SF-36 score. Those who were unemployed had an MMAS score of 60.46 ± 20.21 compared to 51.57 ± 20.45 for those who were employed (Table 3).

**Table 3:** Comparison of the changes in the MMAS, MOS SF-36, and PBAC scores based on education and working status. MMAS = Menorrhagia Multiattribute Scale; MOS SF-36 = Medical Outcomes Study 36-Item Short-Form Health Survey; PBAC = Pictorial Bleeding Assessment Chart

	MMAS score	MOS-SF 36 score	PBAC score
Education			
Illiterate	60.09 ± 21.27	52.03 ± 16.62	155.74 ± 82.25
Primary	55.36 ± 20.19	54.83 ± 15.02	130.96 ± 65.36
Graduate	54.72 ± 20.28	59.24 ± 6.54	141.52 ± 58.94
P-value	0.069	0.007	0.037
Working			
No	60.46 ± 20.21	54.58 ± 13.95	153.98 ± 69.98
Yes	51.57 ± 20.45	56.17 ± 13.78	128.85 ± 70.38
P-value	0.004*	0.221	0.001*

Based on the duration of use, changes in MMAS, MOS SF-36, and PBAC scores were significant (p = 0.001, p = 0.001, and p = 0.005, respectively) (Table 4).

**Table 4:** Comparison of the changes in the MMAS, MOS SF-36, and PBAC scores based on the duration of use. MMAS = Menorrhagia Multiattribute Scale; MOS SF-36 = Medical Outcomes Study 36-Item Short-Form Health Survey; PBAC = Pictorial Bleeding Assessment Chart

	3 months to 1 year	1 to 2 years	2 to 3 years	>3 years	P-value
MMAS score	48.92 ± 19.15	62.39 ± 21.44	59.46 ± 19.97	57.64 ± 20.03	0.001*
MOS SF-36 score	45.61 ± 18.97	57.04 ± 12.85	62.57 ± 5.35	56.95 ± 6.59	0.001*
PBAC score	129.17 ± 70.99	141.09 ± 75.39	138.05 ± 52.98	168.32 ± 75.21	0.005*

**Figure 2:**
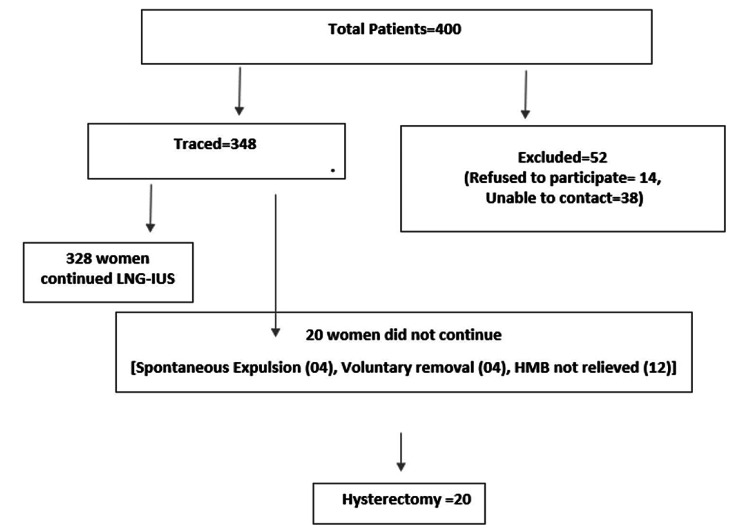
Distribution of participants. LNG-IUS = levonorgestrel intrauterine system; HMB = heavy menstrual bleeding

All 348 patients who were traced and interviewed were divided into four groups according to the duration of use: 92, 92, 76, and 88 participants in the three months to one year, one to two years, two to three years, and more than three years groups, respectively. In the three months to one year group, 13% of the patients’ menorrhagia was not controlled, and they underwent a hysterectomy, 74% developed hypomenorrhea, and 12% became amenorrhoeic. The total number of patients with hypomenorrhea or amenorrhea was maximum (76%) after more than three years. Only 8.7% in the one to two-year use group did not receive relief from heavy bleeding and underwent a hysterectomy. A total of 328 (continuation rate: 94.25%) women continued using the LNG-IUS as their menorrhagia was controlled. The treatment failure rate was 3.44%, as 12 patients’ menorrhagia was not relieved, and they opted for total abdominal hysterectomy. Voluntary expulsion was noted in 1.14% (n = 4) due to pelvic inflammatory disease, and spontaneous expulsion was noted in 1.14% (n = 4) due to an adenomyotic uterus (Figure 2).

**Figure 3:**
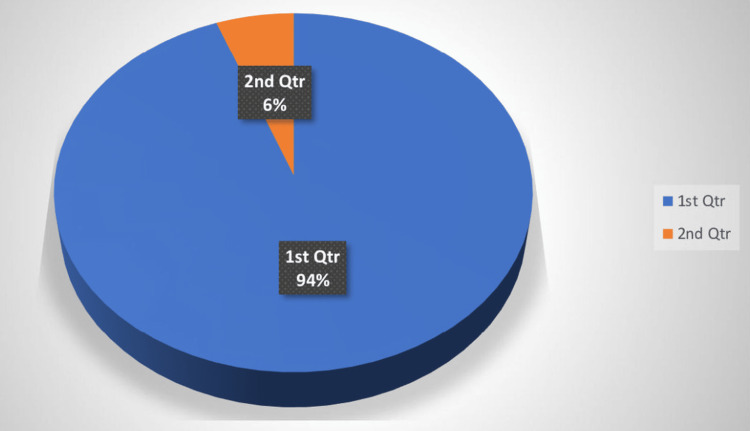
Continuation rate (1) and hysterectomy rate (2).

All patients whose menorrhagia was not relieved or those who reported either spontaneous or voluntary expulsion underwent a hysterectomy (5.74%) (Figure 3).

## Discussion

Globally, studies that objectively measured HMB showed a prevalence of 9-14%, while a subjective assessment showed a prevalence of 20-52% [15]. Therefore, the primary treatment objective is to decrease menstrual blood flow and improve QOL. Previous studies have compared QOL using the LNG-IUS with medical or surgical treatments. However, limited studies have examined long-term improvements in QOL scores in the same cohort of women before and after the insertion of LNG-IUS. Further research is required regarding the efficacy and acceptability of LNG-IUS for the benign etiology of menorrhagia, as it is a potential non-surgical, non-invasive method, although not widely popularized, particularly in India.

Gupta et al. [5] randomly assigned 571 women with menorrhagia to the LNG-IUS or usual medical treatment groups and measured the MMAS and MOS SF-36 scores over a two-year study period. In total, 285 women were randomly assigned to the LNG-IUS group. The MMAS score improved in both treatment groups at six months, one year, and two years. However, the improvement was significantly greater in the LNG-IUS group (p < 0.001). All six domains of the MMAS favored LNG-IUS at all time points. The MOS SF-36 scores also improved significantly from baseline in the LNG-IUS group compared to the usual treatment group in seven of the eight domains. Mental health was the only domain with no significant between-group differences. In our study, all domains of the MMAS and MOS SF-36 improved with LNG-IUS such as in the Effectiveness and Cost-Effectiveness of Levonorgestrel-Containing Intrauterine System in Primary Care Against Standard Treatment for Menorrhagia (ECLIPSE) trial [5]. A hysterectomy was performed in 6% of cases, which was similar to our study, in which it was performed in 5.74% of cases. This finding was also supported by another five-year follow-up randomized trial, in which the hysterectomy rate was significantly lower (3.7%) in the LNG-IUS group compared to the endometrial ablation group [16].

In our study, the change in the mean PBAC scores before and after LNG-IUS insertion was 176.36 and 32.19, respectively (p = 0.001). Both the MMAS and MOS SF-36 scores increased significantly from baseline. These results were consistent with those of Eralil et al. [8]. In their retrospective observational study, 70 women with abnormal uterine bleeding were randomly assigned to the LNG-IUS or usual medical treatment groups and followed up for two years. Both the MMAS and MOS SF-36 scores increased significantly from baseline (p < 0.001).

In a prospective observational study of 572 Asian women from eight countries, 437 were assigned to the LNG-IUS treatment group and 135 to the common medical therapy group [17]. The MMAS scores were recorded at all four visits, namely, baseline, three months, six months, and 12 months. The mean age was 37.8 ± 4.9 years, which was consistent with our study, in which the mean age was 39.74 ± 5.63 years. The baseline mean MMAS score was 41.4 ± 24.5, and at 12 months, it significantly increased to 92.5. In our study, the baseline mean MMAS was 36.73 ± 20.4 before insertion and increased significantly with duration to 93.72 ± 14.62 after insertion.

In our study, over a seven-year study period, four women voluntarily removed their LNG-IUS (removal rate was 1.14%). The reasons were intermenstrual spotting (50%) and pelvic inflammatory disease (50%). The spontaneous expulsion rate due to adenomyosis was 1.14%. The acceptance rate was 94.25%. These findings were consistent with those reported by Wreikat et al. [18], in which after 12 months of Mirena insertion in 120 perimenopausal women, 115 (95.8%) accepted it as a treatment of menorrhagia, and 2.5% had intermenstrual spotting and decided to remove it.

The MMAS and PBAC scores increased significantly (p < 0.004 and p < 0.001, respectively) in the working population. The relief of work embarrassment and the need to carry extra sanitary pads due to menorrhagia may be greater in the working population compared to the non-working population. A significant difference in the MOS SF-36 and PBAC scores was noted between the educated and non-educated groups (p < 0.007 and p < 0.037, respectively). This was because educated people may have less recall bias and a better perception of their general well-being. However, further studies are required to confirm this.

Key messages

LNG-IUS is more effective than medical therapy and a hysterectomy. In addition, it has a greater satisfaction rate. Several studies have compared it with other treatment modalities. However, there is a paucity of literature, especially from Asian countries, regarding the long-term QOL, most studies only examined effects for up to one to two years. This study compared women’s self-perception of improvements in HMB using QOL scores over a seven-year duration.

Strengths and limitations

The major strength of this study was the relatively large study population followed over seven years. In addition to this, validated condition-specific MMAS along with general health perception MOS SF-36 scores were compared. Both qualitative and quantitative measurement tools were used. The major limitation of this study was recall bias, as participants had to recall and answer the duration of bleeding, number of pads, passage of clots, and questions related to their effect on physical, social, emotional, family life, and general well-being prior to LNG-IUS insertion. Another limitation was that this study did not compare the outcomes with other medical or surgical methods and no control group was used.

## Conclusions

Menorrhagia substantially affects day-to-day QOL and has social, physical, emotional, and negative effects on family life. After LNG-IUS use, the mean MMAS and MOS SF-36 scores increased significantly from 36.73 ± 20.40 to 93.72 ± 14.62 and 35.33 ± 6.73 to 90.54 ± 15.89 at follow-up, respectively. The mean PBAC score decreased significantly from 176.36 ± 79.85 to 32.19 ± 63.87. Mean differences were 56.99, 55.21, and 144.16 in the MMAS, MOS SF-36, and PBAC, respectively. Hysterectomy and expulsion rates were significantly low. LNG-IUS decreased excessive bleeding and substantially improved the QOL in all domains. It is a potential treatment option for menorrhea of a benign etiology as it requires less skill, is a non-invasive method, has high efficacy, and is cost-effective.
